# The role of genetic testing in diagnosing Fabry’s disease and its overlapping with cardiomyopathies: a case series

**DOI:** 10.1093/ehjcr/ytae375

**Published:** 2024-08-05

**Authors:** Mustafa Suppah, Hema Narayanasamy, James Nelson, Said Alsidawi

**Affiliations:** Division of Cardiovascular Medicine, Mayo Clinic Hospital, Phoenix, AZ 85054, USA; Division of Cardiovascular Medicine, Mayo Clinic Hospital, Phoenix, AZ 85054, USA; Division of Cardiovascular Medicine, Mayo Clinic Hospital, Phoenix, AZ 85054, USA; Division of Cardiovascular Medicine, Mayo Clinic Hospital, Phoenix, AZ 85054, USA

**Keywords:** Fabry’s disease, Hypertrophic cardiomyopathy, Genetic testing, Heart failure, Case series, Case report

## Abstract

**Background:**

Fabry’s disease, an X-linked lysosomal storage disorder, shares cardiac manifestations with hypertrophic cardiomyopathy (HCM). We underscore the importance of considering Fabry’s disease as a differential diagnosis in HCM patients, highlighting genetic testing’s role in cardiomyopathy evaluation.

**Case summary:**

Three male patients with left ventricular hypertrophy were initially diagnosed with HCM but were later found to have Fabry’s disease through genetic testing. Atypical features such as renal dysfunction and conduction abnormalities raised suspicion. Genetic testing confirmed diagnosis, guiding tailored management.

**Discussion:**

Fabry’s disease poses diagnostic challenges due to its resemblance to HCM. Genetic testing enables precise diagnosis and personalized management, especially in cases with atypical presentations. Early recognition and intervention, facilitated by genetic testing, can improve patient outcomes in Fabry’s disease.

**Learning points**

- To recognize Fabry’s disease as a potential differential diagnosis in patients presenting with hypertrophic cardiomyopathy.
- To identify atypical features associated with hypertrophic cardiomyopathy (HCM) such as renal dysfunction, conduction abnormalities on electrocardiogram, or distinctive echocardiographic findings and expand diagnostic considerations beyond primary HCM.
- To understand the importance of genetic testing in differentiating phenocopies of hypertrophic cardiomyopathy.

## Introduction

Fabry’s disease, an X-linked lysosomal storage disorder caused by mutations in the GLA gene, presents with various clinical manifestations, notably cardiovascular involvement such as Hypertrophic cardiomyopathy (HCM).^1^ Hypertrophic cardiomyopathy is a heart condition characterized by the thickening of the heart muscle, often resulting in symptoms such as chest pain, shortness of breath, fainting, and, in severe cases, syncope and sudden death. Accurate diagnosis is imperative for early management. Genetic testing plays a pivotal role in identifying underlying mutations, aiding in risk stratification and family screening.^2,3^ Distinguishing between primary HCM and phenocopies like Fabry’s disease poses a challenge due to their overlapping clinical and imaging features.^4^

Recent advancements in cardiovascular genetics emphasize the importance of genetic testing in diagnosing HCM and related conditions.^5^ Genetic testing aids in identifying disease-causing mutations, aiding in accurate diagnosis, tailored management strategies and family screening.^6^ Particularly in Fabry’s disease, genetic testing is crucial for confirming diagnosis, especially in cases with atypical presentations or a family history indicative of X-linked inheritance.^7^ This is important to instate treatment with enzyme replacement therapy early in the course of the disease.

Despite the increasing recognition of Fabry’s disease as a potential mimic of HCM, there is a necessity for heightened awareness among clinicians, particularly cardiologists, regarding the diagnostic algorithm and management implications related to this rare lysosomal storage disorder.^8^

This case series aims to highlight the diagnostic challenges posed by Fabry’s disease, emphasize the utility of genetic testing in distinguishing between primary HCM and phenocopies, and stress the importance of early identification for timely initiation of disease-specific therapies.

## Summary figure

**Figure:**
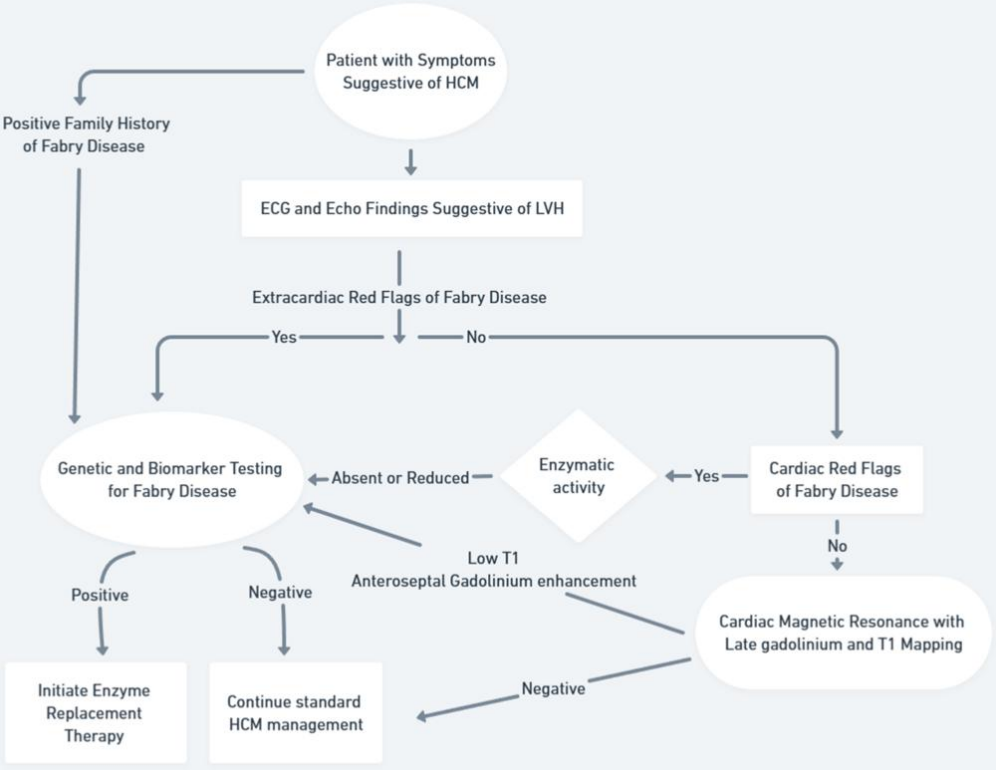


## Case presentation

We present a case series of three male patients referred to our institution with left ventricular hypertrophy (LVH). Despite the initial clinical diagnosis of primary HCM, genetic testing for Fabry’s disease was performed, leading to the identification of a disease-causing variant in the GLA gene, thereby supporting the diagnosis of Fabry’s disease.

### Patient 1

**Figure 1:**
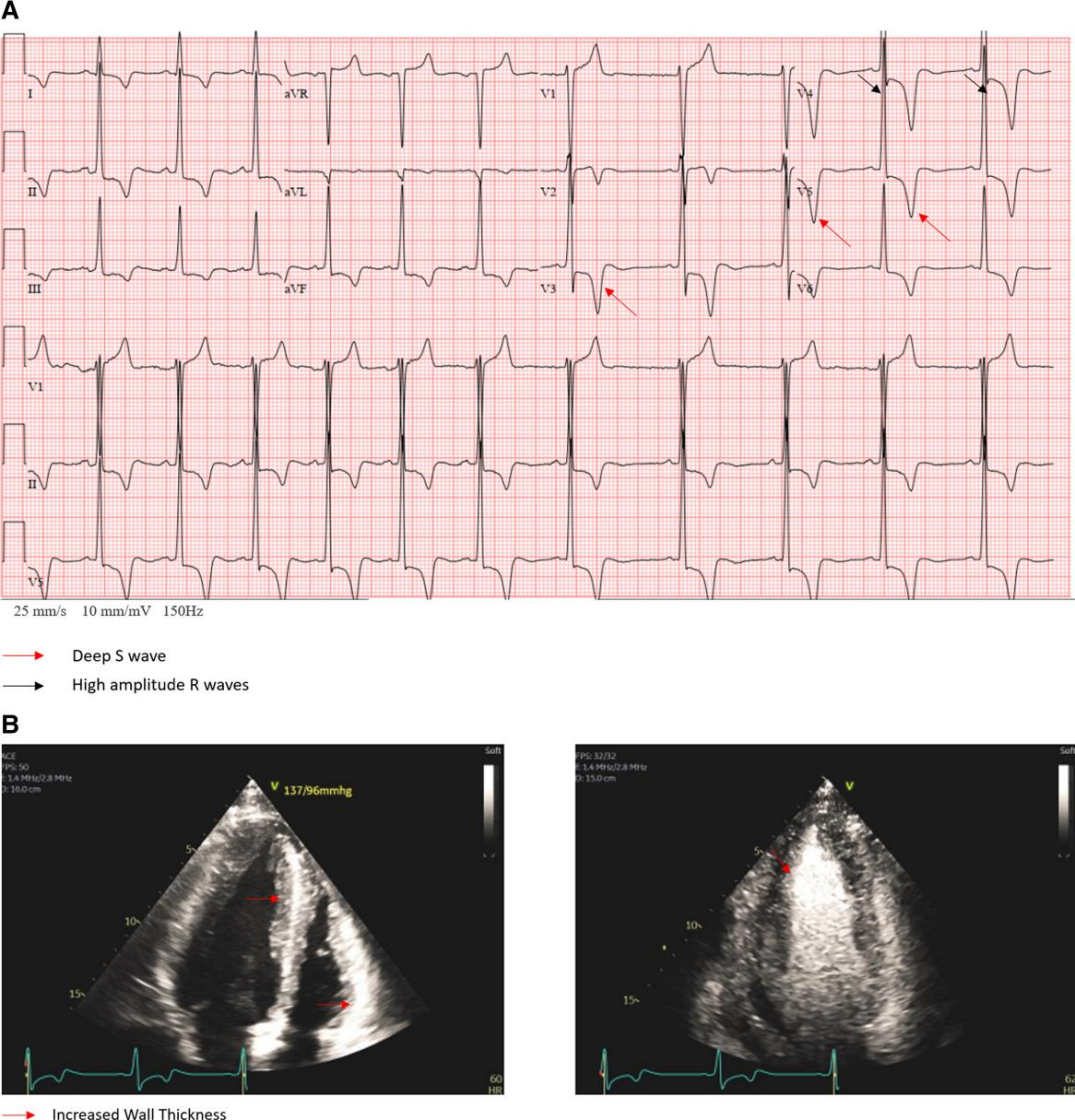
(*A*) Patient 1 electrocardiogram showing findings consistent with left ventricular apical hypertrophy. (*B*) Patient 1 echocardiogram showing left ventricular hypertrophy.

A 38-year-old male with no significant medical history sought a second opinion for symptoms including a pre-syncope event, lightheadedness, and chest tightness. Initial admission to an external facility yielded negative results from an ischaemic workup. However, abnormal findings on electrocardiogram (EKG) (*Figure 1A*) and echocardiogram (*Figure 1B*) consistent with apical hypertrophy prompted his cardiologist to recommend implantable cardioverter-defibrillator placement. Upon referral to our institution for a second opinion, subsequent evaluation revealed apical HCM supported by cardiac magnetic resonance imaging (MRI) and genetic testing, which identified a hemizygous pathogenic variant (c.644A>G) (p.Asn215Ser), confirming the diagnosis of Fabry’s disease, leading to the initiation of enzyme replacement therapy resulting in clinical improvement. This case underscores the importance of comprehensive evaluation and genetic testing in patients with unexplained cardiomyopathies.

### Patient 2

**Figure 2:**
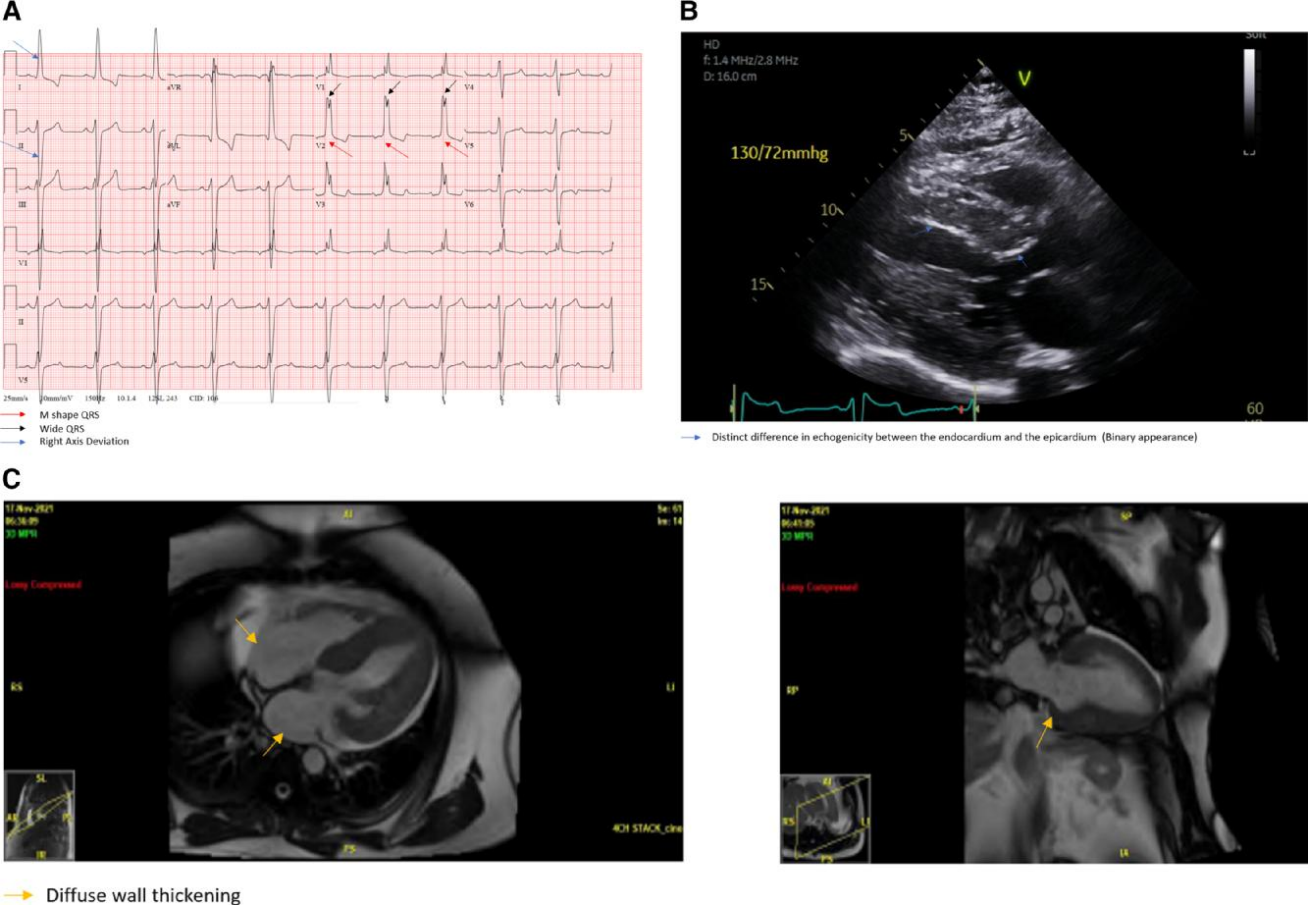
(*A*) Patient 2 electrocardiogram showing right bundle branch block. (*B*) Patient 2 echocardiogram showing significant wall thickness binary appearance of the myocardium. (C) Patient 2 cardiac MRI showing diffuse gadolinium enhancement.

A 62-year-old male with well-controlled hypertension and Type 1 diabetes and a family history of HCM and sudden death presented with worsening fatigue and shortness of breath. Upon evaluation, an EKG revealed conduction abnormalities in the form of right bundle branch block (*Figure 2A*). Echocardiography showed significant wall thickness consistent with HCM (*Figure 2B*). The patient’s left ventricular ejection fraction was measured at 55–60%, with evidence of Grade 1 left ventricular diastolic dysfunction.

Further imaging with cardiac MRI (*Figure 2C*) demonstrated severely increased concentric left ventricular wall thickness, along with diffuse delayed gadolinium enhancement predominantly affecting the basal-mid anterior and inferior septum, indicating areas of scarring consistent with HCM. The calculated scar volume was 8.6%. Left ventricular end-diastolic volume index was measured at 90 mL/m^2^, with a left ventricular mass of 290 g. Right ventricular size and systolic function were found to be normal, with a right ventricular ejection fraction of 58%.

Laboratory findings showed normal kappa and lambda free light chains, with a kappa/lambda free light chain ratio of 1.03, along with slightly elevated N-terminal pro b-tybe natriuretic peptide (NT-proBNP) levels and a low alpha-galactosidase level of 2.68. Genetic testing identified a pathogenic variant in the GLA gene (c.644A>G) (p.Asn215Ser), confirming the diagnosis of Fabry’s disease, with an additional variant of uncertain significance in FHL1. Enzyme replacement therapy was initiated, which resulted in symptom improvement at the 6-month follow-up.

This unique case underscores the importance of a comprehensive diagnostic approach in patients presenting with overlapping features of HCM and Fabry’s disease. Notably, cascade screening in the patient’s family revealed that his brother, who had a history of HCM, also tested positive for Fabry’s disease, emphasizing the familial nature and significance of genetic testing in such cases.

### Patient 3

**Figure 3:**
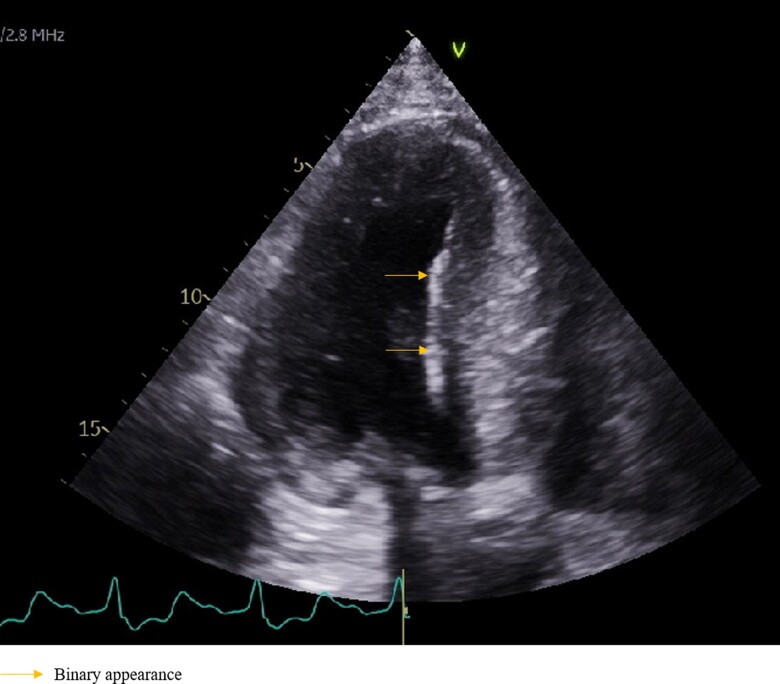
Patient 3 echocardiogram showing binary appearance of the myocardium.

A 64-year-old male with a history of end-stage renal disease and hypertension presented for evaluation for kidney transplantation. During the assessment, he reported symptoms suggestive of HCM, including exertional dyspnea and palpitations. Initial assessment revealed an elevated pro-type b BNP of 3079. Echocardiography showed LVH with a preserved left ventricular ejection fraction of 60–65% but with Grade 2 left ventricular diastolic dysfunction. Normal right ventricular size and function were observed, along with a binary appearance of the myocardium and Valsalva-induced intracavitary gradient/obstruction (*Figure 3*), raising suspicion for HCM. However, further investigation uncovered a complex diagnostic picture.

A kidney biopsy performed as part of the transplantation evaluation revealed features consistent with hypertensive nephropathy but did not lead to a diagnosis of Fabry’s disease at that time.

Cardiac MRI performed as part of the overall assessment, further characterized Patient 3’s cardiac phenotype. The imaging revealed severe asymmetric LVH, with a maximum wall thickness of 3.2–3.3 cm at the basal antero-septum, consistent with HCM. Additionally, evidence of flow acceleration across the left ventricular outflow tract suggested obstruction. Despite these findings, the diagnosis of Fabry’s disease was not initially apparent from imaging alone.

Initial genetic testing for coding variants in GLA, associated with Fabry’s disease, yielded negative results. Despite this, reduced alpha-galactosidase activity on biochemical testing prompted comprehensive genome sequencing. This approach identified a probable intergenic inversion of Exon 4 in GLA, providing a likely genetic explanation for Fabry’s disease.

Patient 3’s case highlights the diagnostic challenges associated with Fabry’s disease, particularly in adults presenting with features mimicking HCM. Despite initial evaluation for kidney transplantation, the comprehensive approach involving clinical evaluation, genetic testing, and imaging ultimately led to a diagnosis of Fabry’s disease. This case underscores the critical role of genetic analysis, including assessment for structural variants, in elucidating the underlying aetiology of cardiomyopathies masquerading as HCM.

## Discussion

The presented case series underscores the intricate diagnostic challenges posed by Fabry’s disease, particularly when it manifests with LVH, resembling HCM. In patients presenting with LVH, the differential diagnosis must extend beyond primary HCM to include phenocopies like Fabry’s disease, necessitating a comprehensive diagnostic approach.

Genetic testing serves as a powerful tool for identifying disease-causing mutations, thereby facilitating precise diagnosis. Furthermore, it assumes a pivotal role in tailoring personalized management strategies and pinpointing individuals at familial risk, thus emphasizing the significance of familial screenings.^5,7^ Particularly in cases where clinical and imaging features deviate from the typical presentation of primary HCM, genetic testing emerges as a crucial tool in differentiating between primary disease and phenocopies like Fabry’s disease.^9^

Patient presentations varied, illustrating the diverse clinical manifestations of Fabry’s disease masquerading as HCM. Notably, clinical suspicion was heightened in cases presenting with atypical features such as renal dysfunction, conduction abnormalities on EKG, or a binary appearance on echocardiogram.^5,9^ Early recognition of these red flags prompted further investigation, leading to the eventual diagnosis of Fabry’s disease through genetic testing.

Moreover, the inclusion of genetic testing in the diagnostic algorithm not only aids in accurate diagnosis but also holds significant implications for patient management. Early identification of Fabry’s disease facilitates the initiation of disease-specific therapies such as enzyme replacement therapy, potentially mitigating disease progression and improving clinical outcomes.^7,8^

## Conclusion

In conclusion, this case series underscores the importance of considering Fabry’s disease as a potential mimic of HCM, particularly in patients presenting with atypical features. Genetic testing emerges as a cornerstone in the diagnostic evaluation of LVH, enabling timely identification of underlying genetic disorders like Fabry’s disease. By integrating genetic testing into clinical practice, clinicians can enhance diagnostic accuracy, optimize patient management, and ultimately improve patient care and outcomes.

As authors of this case series, we confirm that appropriate informed consent has been obtained from all patients included. We affirm compliance with the guidelines established by the Committee on Publication Ethics (COPE) regarding patient confidentiality, anonymity, and consent for publication. We assure that patient identities have been adequately protected, and any identifiable information has been anonymized or removed to ensure privacy.

## Lead author biography


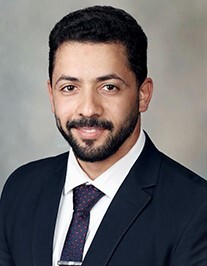


Dr Mustafa Suppah is an internal medicine resident at Creighton University-Phoenix and a research scholar at Mayo Clinic Arizona with a special interest in cardiovascular diseases. He graduated from Ain Shams Medical School in Cairo, Egypt, and then pursued a research fellowship training at Mayo Clinic Arizona and currently doing his internal medicine residency in Creighton University-Phoenix. Dr Suppah has presented his research at numerous national and international conferences, including the American Heart Association Scientific Sessions, ACC, TCT, and TVT. Their current research focuses on improving diagnostic strategies and personalized management approaches for patients with inherited cardiomyopathies.

## Data Availability

The data used to support the findings of this case series are available from the corresponding author upon request. However, due to privacy and ethical considerations, some restrictions may apply to the availability of patient-specific data. Access to patient data will be granted in accordance with institutional policies and regulations governing patient privacy and confidentiality.
